# Quantum error channels in high energetic photonic systems

**DOI:** 10.1038/s41598-024-60472-1

**Published:** 2024-04-27

**Authors:** B. C. Hiesmayr, W. Krzemień, M. Bała

**Affiliations:** 1 University of Vienna, Faculty of Physics, Währingerstrasse 17, 1090 Vienna, Austria; 2 High Energy Physics Division, National Centre for Nuclear Research, Andrzeja Soltana 7, 05-400 Otwock, Swierk, Poland; 3 Department of Complex Systems, National Centre for Nuclear Research, Andrzeja Soltana 7, 05-400 Otwock, Swierk, Poland

**Keywords:** Quantum information, Theoretical physics

## Abstract

In medical applications—such as positron emission tomography (PET)—511 keV photons that experience Compton scattering are studied. We present a consistent framework based on quantum error-correction channels—intensively studied in quantum computing—to fully describe the quantum information-theoretic content of high energetic photons undergoing Compton scattering, characterized by the Klein–Nishina formula in unoriented matter. In this way, we can predict the expected spatial distribution of two or more, pure or mixed, polarization entangled or separable photons. This framework allows us to characterize the accessible and inaccessible information for different parameter ranges. It also answers the question of how to describe successive multi-photon scattering. In addition our formalism provides a complete framework for dealing with single and all multi-partite errors that can occur in the propagation, providing the basis for modeling future dedicated experiments that will then have applications in medicine, such as reducing errors in PET imaging or exploring possibilities for quantum-based diagnostic indicators.

## Introduction

In the domain of low energetic systems such as e.g. currently intensively studied for quantum computing^1^ Kraus representations of error channels are one way to characterize errors. In this paper we show that and how the error correction theory can be extended to the domain of high energetic photons for Compton scattering events based on the Klein–Nishina formula. Typically errors that entangle portions of the system of interest with the environment appear to be non-unitary quantum operations, i.e. one can no longer assume or simplify the system of interest as a pure state, hence our knowledge of the state of interest is not complete. Generally, one assumes that the mathematical object to cover any available information of a quantum system is given by a semi-positive-definite Hermitian density operator $\rho =\sum _j p_j\; |\psi _j\rangle \langle \psi _j|,$ representing a statistical mixture of different states $|\psi _j\rangle$ that occur with probabilities $p_j$ obeying in general $\sum _j p_j=1$. However, as is well known the decomposition is not unique. Only if one probability equals one, we have a pure state, complete information of the system is available. Generally one assumes that an allowed dynamic of a physical system during some time interval $\Delta t$ transforms $\rho$ into a semi-positive-definite Hermitian matrix $\rho ^{\prime}$ and this dynamic is identified by completely-positive trace-preserving maps^2^. Therefore, in the so-called Kraus representation, one can describe a transformation by

1 $$\begin{aligned} \rho ^{\prime}=\rho (t+\Delta t)=\sum _l K_l\; \rho (t)\; K_l^\dagger \end{aligned}$$

with the completeness relation $\sum _l K_l^\dagger K_l=\mathbbm {1}$, which follows from the invariance of the trace under cyclic permutations of the operators. One important feature of the Kraus representation is that these operators are not unique, i.e. defining $F_l=\sum _k U_{lk}\; K_k$ it follows $\sum _l K_l \rho (t) K_l^\dagger =\sum _l F_l \rho (t) F_l^\dagger$ with *U* being a unitary transformation. This property will be the key to solve fundamental issues in this high energetic systems, or differently stated to test if the fundamental laws of quantum theory also apply in this energy regime.

**Figure 1:**
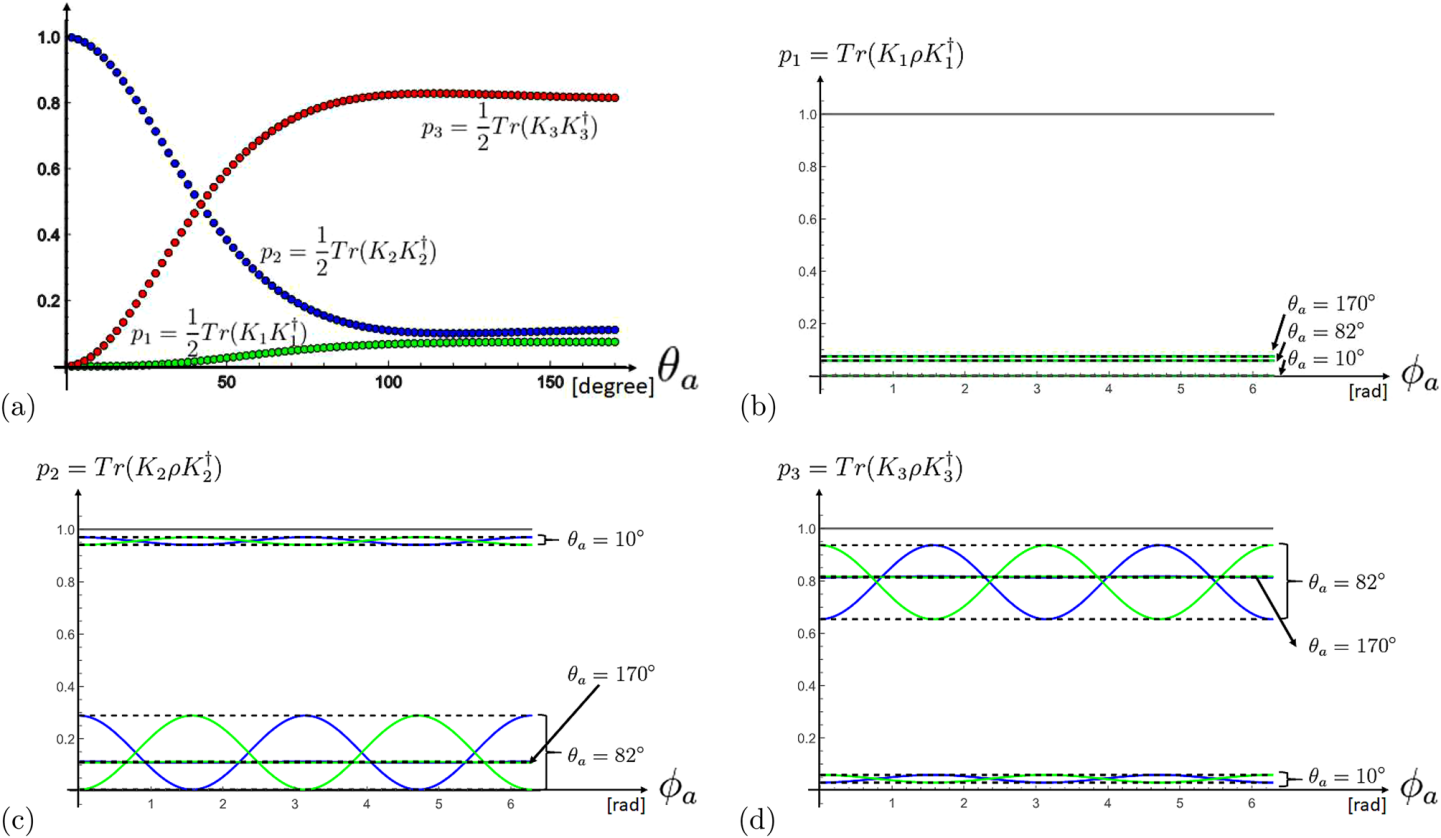
These graphics show the result of the three probabilities $p_1=Tr(K_1 \rho K_1^\dagger )$, $p_2=Tr(K_2 \rho K_2^\dagger )$ and $p_3=Tr(K_3 \rho K_3^\dagger )=1-p_1-p_2$ deduced from the Kraus operators for (**a**) unpolarized states $\rho =\frac{1}{2}\mathbbm {1}$ and (**b**)–(**d**) different pure states. In (**b**)–(**d**) the blue and green curves corresponds to $|H\rangle$ and $|V\rangle$ polarized states (with respect to the chosen coordinate system $(\theta _s=0,\phi _s=0)$), respectively. The plots show the result for the scattering angles $\theta _a=10^\circ ,82^\circ ,170^\circ$, characterizing the three different regions of CKN events. The dashed lines correspond to pure states optimized over unitaries (more details are given in the “Appendix 1”).

In error correction theory^3–7^ and when working in the interaction picture, the set of Kraus operators $\{K_1,K_2,\dots \}$ is interpreted as the transformation of a state under various errors. So for instance, a set of errors could be a qubit suffering from bit flip $K_1=\sqrt{p_1}\, \sigma _1$ and a phase flip $K_2=\sqrt{p_2}\,\sigma _3$ and therefore the case that no error occurs is given by $K_3=\sqrt{1-p_1-p_2}\, \mathbbm {1}_2$. Typically, one interprets the expectation value

2 $$\begin{aligned} \langle K_l^\dagger K_l\rangle =Tr\left( K_l\;\rho (t)\;K_l^\dagger \right) =p_l \end{aligned}$$

as the probability that the *k*th error will occur or has occurred. In error correction theories those probabilities are e.g. exploited to construct logical codewords by the Knill-Laflamme condition^8^.

In this paper, we consider the Compton scattering process of 511keV photons, initiated by the propagation of photons through a medium described by the Klein–Nishina formula^9^, along the theoretic framework described above. In the following, we denote this process as a Compton–Klein–Nishina (CKN) scattering event.

The proper modelling of the CKN processes is important in developing new medical imaging applications for positron emission tomography (PET). PET is a non-invasive medical imaging technique used commonly in diagnostics enabling metabolic imaging of pathological tissues. The radiopharmaceutical (typically fluorodeoxyglucose, FDG) is administrated to the patient and absorbed by organs and tissues. The positron originating from the $\beta +$ decay of the radionuclide annihilates with an electron from the patient’s body, and in consequence, two back-to-back photons are emitted. The set of the photon pairs (coincidences) registered within the time window by the scanner serves as input data for the iterative algorithm (e.g. Maximum Likelihood Expectation Maximization) that reconstructs the original distribution of the activity in the patient’s body forming a 3-D image that is further analysed by the medical doctors.

For decades PET imaging was based on the two-photon model mostly omitting the details of the quantum-mechanical description of the process. Indeed direct electron-positron annihilation happens only in about $40\%$ of the cases, otherwise, the positron and electron form a quasi-stable atom called positronium. The positronium atom can be in either a symmetric or antisymmetric spin state so that it decays into an even or an odd number of photons. This means that three photon events also occur, but are not exploited in standard hospital devices. Interestingly, depending on the angles between the photons different entangled states are formed, and predicted for nearly all angles to be genuine multipartite entangled, i.e. the three-photon events are predicted to exist in the strongest form of entanglement (for more details see Ref.^10^). For the two-photon events a maximally entangled state is predicted that we also discuss in this contribution.

Recently, it was proposed to exploit the properties of the positronium such as e.g. its lifetime or complementary information about the processes at the cellular level in the patient’s body^11,12^. This opens new possibilities for the next generation of PET scanners. Also, other properties of the positronium system might be of medical interest. One of the consequences of positronium formation is that the pairs (or triples) of photons decaying from a defined quantum state can carry the correlated polarization information. It turns out that the high-energy photon polarization can be partly determined via measuring the scattering angles of the photon process and this paper presents its foundations from the quantum information theoretic view via quantum channels build up by Kraus operators.

The possible applications of the photon-pair quantum correlation in the context of PET medical imaging are twofold. First, the quantum correlation can be exploited to reduce the unwanted false coincidences^13–15^, that deteriorates the final PET image quality. The false coincidences consist of pairs in which the two registered photons do not originate from the same origin (so-called randoms), or at least one of the photons is deflected via the Compton scattering in the patient’s body before reaching the scanner. The random coincidences are by definition uncorrelated. A more complex situation is the question of what happens with the quantum correlations after one of the photons undergoes the Compton scattering in the patient’s body. The key idea is that the measurement of the polarization degree of freedoms would allow for better discrimination of the unwanted pairs and lead to improved contrast.

The second more exploratory approach consists of the analysis of the the quantum correlations carried by the photons as a new type of diagnostic indicator, that might bring complementary information to the biological processes of the patient’s body^10,16^. Although, currently not possible to achieve technically, this approach seems like an interesting path to investigate.

The measurements of the polarization require the detection of the photon scattering in the scanner device. The current standard PET devices operating in hospitals consist of inorganic crystals like BGO or LYSO that record the 511 keV photons through the photoelectric effect. However, several novel setups have been proposed. E.g. a novel plastic-based PET device (called J-PET) is currently being built on the basis of new technology (e.g. Refs.^17^) and is already undergoing its first clinical tests. Also, so-called Compton PET systems are being investigated (Ref.^14,18^) as well as other setups capable of polarization measurement (e.g. Ref.^15^).

To study the underlying physics exploited in the PET scenario based on plastic scintillators one needs to understand the Compton–Klein–Nishina scattering of single photons (“Section Kraus formalism for single Compton–Klein–Nishina scattered photons”). In “Section Compton–Klein–Nishina scattering as a quantum channel” we formulate this interaction as a Kraus channel illustrating the similarities to the error correction theory. In the next step one needs to apply the formalism to two photons. This is not straight forward since the two photons can be in an entangled state. We solve this conceptual problem by the Kraus formalism given in “Section Scattering of bipartite photons and their losses to the environment”. In the following up we present particular results of our consistent formalism and conclude with a discussion (“Section Discussion and conclusion”).

## Methods

Here we show firstly how the Compton–Klein–Nishina formula^9^ can be reformulated by two Kraus operators analogous to Ref.^16^, however, we take the overall normalization into account. This allows us to find the third Kraus operator to complete the completeness relation and therefore study which information is transferred to the environment.

### Kraus formalism for single Compton–Klein–Nishina scattered photons

In Ref.^16^ the authors have presented a pseudo Kraus representation leading to the same result as the Klein–Nishina formula^9^ when summing over final states. It is called pseudo Kraus representation, because the two Kraus operators do not satisfy the completeness relation. In the following, we present Kraus operators satisfying the completeness relation from which a full description of the CKN scattering events can be deduced, this result is relevant for improving e.g. positron-emission-tomograph (PET) imaging^14,15,19–26^.

Different to Ref.^16^ we define the Kraus operators with an overall normalization of the scattering process by

3 $$\begin{aligned} K_1= & {} \frac{\left( \frac{(\sin \theta _s \sin \theta _a \cos (\phi _s-\phi _a)+\cos \theta _s \cos \theta _a-1)^2}{-\sin \theta _s \sin \theta _a \cos (\phi _s-\phi _a)-\cos \theta _s \cos \theta _a+2}\right) ^{3/2}}{\sqrt{2} (-\sin \theta _s \sin \theta _a \cos (\phi _s-\phi _a)-\cos \theta _s \cos \theta _a+1)^2} \left( \begin{matrix} 1&{} 0 \\ 0 &{} 1 \\ \end{matrix} \right) \end{aligned}$$

4 $$\begin{aligned} K_2= & {} \left( \begin{array}{ll} \frac{1}{(\cos \theta _s \cos \theta _a-2) {\text {sec}} (\phi _s-\phi _a)+\sin \theta _s \sin \theta _a} &{} -\frac{i \cos \theta _s \sin (\phi {\text {s}}-\phi _a)}{\sin \theta _s \sin \theta _a \cos (\phi _s-\phi _a)+\cos \theta _s \cos \theta _a-2} \\ \frac{i \cos \theta _a \sin (\phi _s-\phi _a)}{\sin \theta _s \sin (\theta (a)) \cos (\phi _s-\phi _a)+\cos \theta _s \cos \theta _a-2} &{} -\frac{\cos \theta _s \cos \theta _a \cos (\phi _s-\phi _a)+\sin \theta _s \sin \theta _a}{\sin \theta _s \sin \theta _a \cos (\phi _s-\phi _a)+\cos \theta _s \cos \theta _a-2} \\ \end{array} \right) . \end{aligned}$$

Here the angles $(\theta _s,\phi _s)$ describe the chosen coordinate systems and $(\theta _a,\phi _a)$ the respective change of the propagation direction of the photon undergoing the CKN scattering. The distribution in real space is then given by the probability

5 $$\begin{aligned} p(\theta _s,\phi _s,\theta _a,\phi _a;\rho )=\sum _{i=1}^2 Tr(K_i\;\rho \; K_i^\dagger ), \end{aligned}$$

where $\rho$ is the density matrix defining the polarization of the source with respect to the chosen coordinate system $(\theta _s,\phi _s)$. This formula is identical to the scattering cross section of 511 keV photons undergoing a Compton scattering e.g. in a plastic scintillator or in crystals. Here one assumes that the scattering at such high energies as 511 keV is described in a good approximation independent of the electron momentum distributions, the orientations of the electron spins and the nuclear spins in the target materials. The qualitative validity of this formula has been shown in several experiments^14,15,19–26^.

### Compton–Klein–Nishina scattering as a quantum channel

Usually, a system of interest that has no unitary evolution is modelled by adding the environment such that it becomes a closed system and evolves according to the Schrödinger equation. So the question is whether we can find one or more Kraus operators that satisfy the completeness relation.

Indeed, we can find such a completeness relation. A third Kraus operator doing the job, i.e. $\sum _{l=1}^3 K_l^\dagger K_l=\mathbbm {1}$, can be found (for which we have chosen $\theta _s=0,\phi _s=0$)

6 $$\begin{aligned} K_3= & {} \frac{1}{\sqrt{2}} \sqrt{1-A-\sqrt{(1-A)^2-B^2}} \left( \begin{array}{cc} \frac{\left( 1-A-B \cos (2 \phi _a)+\sqrt{(1-A)^2-B^2}\right) }{B} &{} i\sin (2 \phi _a) \\ i\sin (2 \phi _a) &{} -\frac{\left( 1-A+B \cos (2 \phi _a)+\sqrt{(1-A)^2-B^2}\right) }{B} \\ \end{array} \right) \end{aligned}$$

with

7 $$\begin{aligned} A= & {} \frac{15 \cos \theta _a-6 (\cos (2 \theta _a)+3)+\cos (3 \theta _a)}{8 (\cos \theta _a-2)^3}\nonumber \\ B= & {} \frac{\sin ^2\theta _a}{2 (\cos \theta _a-2)^2}. \end{aligned}$$

In Fig. 1a we have plotted the probabilities $p_1=Tr(K_1 \rho K_1^\dagger ),p_2=Tr(K_2 \rho K_2^\dagger ),p_3=Tr(K_3 \rho K_3^\dagger )=1-p_1-p_2$ as a function of the Compton scattering angle $\theta _a$ for a completely unpolarized photon ($\rho =\frac{1}{2} \mathbbm {1}$) for a fixed coordinate system $(\theta _s=0,\phi _s=0)$. It is found that the first error, corresponding to $K_1$, increases slowly with the Compton scattering angle, in contrast to the second error, corresponding to $K_2$, which decreases rapidly with increasing Compton scattering angle. The third error, corresponding to $K_3$, may be interpreted as the loss to the environment (i.e. electrons in this case). For scattering angles larger than $82^\circ$, a maximum loss of about $80\%$ to the environment is reached. Unfortunately, also the second error, corresponding to $K_2$, decreases then, which is the one sensitive to the polarisation of the photon. Thus this illustrates the three known regimes of the Compton–Klein–Nishina scattering events: small scattering angles (we choose in the following $\theta _a=10^\circ$), large scattering angles ($\theta _a=170^\circ$) and the optimal angle for deducing polarization $\theta _a=82^\circ$.

In Fig. 1b–d, $p_1=Tr(K_1 \rho K_1^\dagger ),p_2=Tr(K_2 \rho K_2^\dagger ),p_3=Tr(K_3 \rho K_3^\dagger )=1-p_1-p_2$ for pure initial states are plotted as a function of $\phi _a$ for three different Compton scattering angles $\theta _a=10^\circ , 82^\circ , 170^\circ$. The blue and green oscillating curves are the results for an initial $|H\rangle$ or $|V\rangle$ state (with respect to a fixed coordinate system $(\theta _s=0,\phi _s=0)$). The upper and lower bounds show the unitary optimization over all pure states, i.e., $p_j=\max _{U}Tr(K_j\;U |H\rangle \langle H| U^\dagger K_j)$ and $p_j=\min _{U}Tr(K_j\;U |H\rangle \langle H| U^\dagger K_j)$, respectively. We observe that the range of the probability for Compton scattering angles around $82^\circ$ is quite large compared to smaller or larger scattering angles. This is because at the extremal values the term in front of the oscillation, the fringe visibility, is quite low, and explains why this is an optimal angle for deducing the polarization.

Interestingly, although at a scattering angle of about $82^\circ$ the information loss to the environment $p_3$ is quite high, this is the best region to validate the oscillation of a polarized photon, in contrast to the region of small Compton scattering angles, where both, the information losses to the environment $p_3$ are small and the sensitivity to the oscillation is quite small. For large scattering angles, we observe large information losses to the environment and low sensitivity to the oscillation. In summary, the quantum information in bipartite Compton–Klein–Nishina scattering is a tradeoff between the loss of information to the environment and the sensitivity to the polarization introduced oscillation.

## Results

Here, we apply our formalism to bipartite systems with and without further scattering, which allows us to predict the possible processes allowed by the quantum theory.

### Scattering of bipartite photons and their losses to the environment

**Figure 2:**
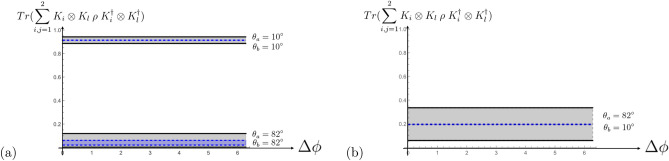
These graphics show the result of the probabilities observed in experiment for pure bipartite states $\rho$ for different scattering scenarios (**a**) both photons scatter either under $\theta _a=\theta _b=10^\circ$ or $\theta _a=\theta _b=82^\circ$ and (**b**) one photon under $\theta _a=82^\circ$ and the other one under $\theta _b=10^\circ$ in dependence of $\Delta \phi =\phi _a-\phi _b$ (with $\phi _b=0$). The bold lines bound the grey area which is the region for any initial separable states, whereas the dashed blue lines bound the region for any initial entangled state (more details can be found in “Appendix 1”). The loss of information is high if the scattering angles are both not small.

In experiments, one uses sources that produce a photon pair with energy 511 keV, at which each photon of the pair scatters. It is still open which initial polarization state of a photon pair has to be assumed for typical sources, most experiments assume that the maximally entangled Bell state $|\psi ^+\rangle =\frac{1}{\sqrt{2}}\left( |HV\rangle +|VH\rangle \right)$ is the relevant one. However, as we show below also a separable state, i.e. $\rho _{\text {mixed}}=\frac{1}{2}\left( |\psi ^+\rangle \langle \psi ^+|+|\psi ^-\rangle \langle \psi ^-|\right)$ with $|\psi ^-\rangle =\frac{1}{\sqrt{2}}\left( |HV\rangle -|VH\rangle \right)$ leads to the same probabilities. Therefore it can be doubted whether the source produces correlations via entanglement.

According to the postulates of quantum theory and assuming the formalism for a single photon CKN scattering is valid the spatial probability of a photon pair each undergoing a CKN scattering should be given by

8 $$\begin{aligned}{} & {} p_\text {double scattering}(\theta _s,\phi _s,\theta _a,\theta _b,\phi _a,\phi _b,\rho )\nonumber \\{} & {} \quad = Tr\left( \sum _{i,j=1}^{2} K_i\otimes K_l\;\rho \; K_i^\dagger \otimes K_l^\dagger \right) \end{aligned}$$

for any initial two-particle state $\rho$, being either pure or mixed and either being separable or entangled. The spatial distribution has been recorded e.g. for a source of $^{22}\text{ Na }$ radioisotope emitting positrons, which interact with an electron and then likely form positronium atoms, which subsequently decay into two or three photons^10,19^. These photon pairs seem to qualitatively behave according to the above formula^21^. Researchers currently also develop new sources with different quantum properties^27^.

In Fig. 2 we plotted the results based on the formula (8) for different scattering angles and pure initial states. If the initial state is entangled we observe that the region of possible values is far more restricted than if the initial state is separable.

Let us emphasize that the probability given by formula 8 does not change by choosing for $\rho$ the states $|\psi ^{\pm }\rangle$ and $\rho _{\text {mixed}}$. This is because in this case, the probability $p_\text {double scattering}$ becomes effectively dependent on $\Delta \phi =\phi _b-\phi _a$. It also reflects the Bose symmetry, and is not sensitive to the sign in the superposition. More details to this can be found in Ref.^16^. The loss to the environment is now given by the sum of all contributions including at least one $\mathcal {K}_3$, quantifying in detail what kind of loss in each case is relevant.

**Figure 3:**
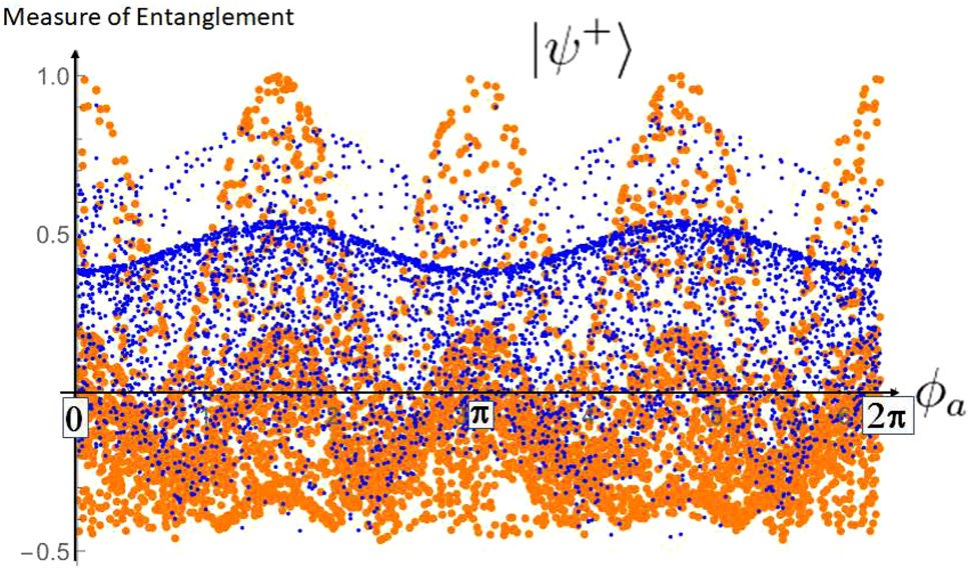
This graphic shows the result of the value of a measure of entanglement (here, the one of Ref.^29,30^ and explicitly given in the “Appendix 2”) of 5000 states generated via $\rho =\sum _{i,j=1}^3 K_i\otimes K_j |\psi ^+\rangle \langle \psi ^+| K_i^\dagger \otimes K_j^\dagger$ (small blue dots) or $\rho _{\text {accessible}}=\sum _{i,j=1}^2 K_i\otimes K_j |\psi ^+\rangle \langle \psi ^+| K_i^\dagger \otimes K_j^\dagger / Tr(\sum _{i,j=1}^2 K_i\otimes K_j |\psi ^+\rangle \langle \psi ^+| K_i^\dagger \otimes K_j^\dagger )$ (big orange dots) for random generated Compton scattering angles $\theta _a,\theta _b$ and random $\phi _a\in [0,2\pi ]$ (plotted at the *x*-axis) with $\phi _b=0$. It proves that the CKN double scattering process is an entanglement breaking channel.

### One photon in the pair scatters a second time

Now let us investigate the situation that a photon pair is generated and one of the photons undergoes a CKN scattering a second time. Using the idea that the CKN scattering process introduces either $K_1$ or $K_2$ or both errors, we could think of two different scenarios, namely either all intermediate states are included $\sum _{c,a,b=1}^{2} K_c.K_a\otimes K_b\;\rho \; K_a^\dagger K_c^\dagger \otimes K_b^\dagger$ or only a particular “error” $K_z$ happens $\sum _{c,b=1}^{2} K_c.K_z\otimes K_b\;\rho \; K_z^\dagger K_c^\dagger \otimes K_b^\dagger$. On the other hand, we know that the representation of the Kraus-operators is not unique, i.e. $\sum _l K_l \rho (t) K_l^\dagger =\sum _l F_l \rho (t) F_l^\dagger$ with $F_j=\sum _k U_{jk}\; K_k$. In both discussed cases the *U* does not cancel out via permutations under the trace, in contrast to the double scattering case (8). Therefore, we conclude that the CKN scattering process has to be considered a measurement-like process. Thus the photon after the scattering is described by the reduced state and then undergoes a CKN scattering process. This gives probabilities that do not depend on the choice of the Kraus operators

$$\begin{aligned} p_{\text {scattered}}=\sum _{c,a,b=1}^{2} Tr\left( \tilde{K}_c Tr_B \left( K_a\otimes K_b\;\rho \; K_a^\dagger \otimes K_b^\dagger \right) \tilde{K}_c^\dagger \right) . \end{aligned}$$

Note, here $Tr_B$ denotes the trace over all degrees of Bob’s (i.e. the second) system, namely the photon that does not scatter a second time. Again the sum over *c* is necessary, since we assume no orientations in the scattering material. Consequently, this is the experimental result to be expected if our assumptions above are valid. Note that after a scattering the energy may be reduced, thus one has to adapt the corresponding Kraus operators for those energies.

### Errors in the propagation

Having established a quantum channel to describe fully the CKN scattering process, we also established a theoretical framework to handle potential errors. Here, we have now different possibilities such as an error occurring to one photon or simultaneously both photons and so on. If the error is of a locally unitary form, i.e. $U_1\otimes U_2$, no difference is expected for the probability $p_\text {double scattering}$ nor $p_{\text {scattered}}$ due to the permutation symmetry under the trace. Thus only a global unitary error or a non-unitary interaction in one subsystem introduces deviations. Whether such experimental settings with high enough statistics are achievable must be clarified by further studies out of the scope of this contribution.

### Property of the CKN error channel

In Fig. 3 we show that the channel describing the double scattering of the pair is entanglement breaking^28^ if the initial state is assumed to be a Bell state. On the other hand starting with the separable mixed state $\rho _{\text {mixed}}$ for all values, we found no revival of entanglement. Counter-intuitively, the information in principle accessible via experiments (big orange dots) shows higher values of entanglement than the corresponding information for the state including the environment (small blue dots). This is due to the entanglement monogamy^31^, i.e. the fundamental property that entanglement cannot be freely shared between arbitrarily many parties.

## Discussion and conclusion

For a long time it was unclear how to treat Compton scattering at high energies from the point of view of quantum information theory: Is the scattering a measurement process? What information about the polarization is revealed? What is the state of the photon after another scattering process? How do errors propagate? We have answered these questions by presenting a consistent framework that treats scattering as a quantum error channel. This theoretical framework will be the starting point for the development of a Monte Carlo simulator that will finally allow comparison with experimental data. This in turn will provide the basis for improving, for example, PET imaging based on the decay of positronium by improving the filtering and classification of scattering events.

## Data Availability

All data generated or analysed during this study are included in this published article.

## Appendix 1: Explicit formulae

Here we give the explicit formulae for different initial states of equation (8) describing the double scattering events. More details are discussed in Ref.^16^.

For an initial maximally entangled state $|\psi ^+\rangle =\frac{1}{\sqrt{2}}\left\{ |H V\rangle -|V H\rangle \right\}$ or the separable mixed state $\rho _{\text {mixed}}=\frac{1}{2} |HV\rangle \langle VH|+\frac{1}{2} |VH\rangle \langle HV|$ we choose the coordinate system such that the two Compton scattering angles $\theta _a,\theta _b$ are the opening angles between the line spanned between the two back-to-back propagating photons (corresponding to $\theta _s=0$)

9 $$\begin{aligned} p_\text {double scattering}(|\psi ^+\rangle )= & {} p_\text {double scattering}(\rho _{\text {mixed}})\nonumber \\= & {} \frac{(15 \cos \theta _a-6 (\cos (2 \theta _a)+3)+\cos (3 \theta _a)) (15 \cos \theta _b-6 (\cos (2 \theta _b)+3)+\cos (3 \theta _b))}{64 (\cos (\theta _a)-2)^3 (\cos \theta _b-2)^3}\nonumber \\{} & {} \quad \frac{-16 \sin ^2\theta _a (\cos \theta _a-2) \sin ^2\theta _b (\cos \theta _b-2) \cos (2 (\phi _a-\phi _b))}{64 (\cos (\theta _a)-2)^3 (\cos \theta _b-2)^3}\nonumber \\= & {} p_\text {double scattering}(\theta _a,\theta _b,\phi _a-\phi _b), \end{aligned}$$

where $|H\rangle ,|V\rangle$ are chosen in a computational basis with respect to the change given by the Compton scattering angle $\theta _a$. The angle $\phi _s$ was chosen such that one matches the polarisation of the right and left photon and respects the Bose symmetry (see also the appendix of Ref.^16^). The reason why both states give the same result lies in the fact that for those basis choices the off-diagonal states do vanish due to the structure of the Kraus operators.

The pure state $|HV\rangle$ and $|VH\rangle$ compute to

10 $$\begin{aligned} p_\text {double scattering}(|HV\rangle )= & {} -\frac{\sin ^4\left( \frac{\theta _a}{2}\right) (7 \cos \theta _b-5 \cos (2 \theta _b)+\cos (3 \theta _b)-7)}{2 (\cos \theta _a-2)^3 (\cos \theta _b-2)^3}\nonumber \\{} & {} +\frac{(7 \cos \theta _b-5 \cos (2 \theta _b)+\cos (3 \theta _b)-7) \left( \cos ^2\theta _a \sin ^2(\phi _a-\phi _b)+\cos ^2(\phi _a-\phi _b)\right) }{4 (\cos \theta (a)-2)^2 (\cos \theta _b-2)^3},\nonumber \\ p_\text {double scattering}(|VH\rangle )= & {} \frac{\sin ^4\left( \frac{\theta _a}{2}\right) (-8 \cos \theta _b+\cos (2 \theta _b)+11)}{2 (\cos \theta _a-2)^3 (\cos \theta _b-2)^3}\nonumber \\{} & {} +\frac{(8 \cos \theta _b-\cos (2 \theta _b)-11) \left( -2 \sin ^2(\theta _a) \cos (2 (\phi _a-\phi _b))+\cos (2 \theta _a)+3\right) }{16 (\cos \theta _a-2)^2 (\cos \theta _b-2)^3}. \end{aligned}$$

In Fig. 2 we present the results for particular choices of $\theta _a,\theta _b$ and different initial states, i.e. the maximal and minimal value over all possible pure separable or pure entangled states,namely

11 $$\begin{aligned} p_\text {double scattering}(\theta _a,\theta _b,\phi _a-\phi _b)=\text {opt}_{U_1,U_2} Tr\left( \sum _{i,j=1}^{2} K_i\otimes K_l\;U_1\otimes U_2\;\rho \;U_1^\dagger \otimes U_2^\dagger \; K_i^\dagger \otimes K_l^\dagger \right) , \end{aligned}$$

where $U_1, U_2$ are unitary operators and for $\rho$ we chose without loss of generally the separable state $|HV\rangle$ or the entangled state $|\psi ^+\rangle$. Since local unitary operations conserve the entanglement property, i.e. separable states are transformed to separable states and entangled states to entangled states. In this way we could find the region of possible values for given $\theta _a,\theta _b$ by assuming either initial separable or entangled states, visualized in Fig. 2.

## Appendix 2: Measure of entanglement

The quasipure approximation^29^ $C_{qp}$ provides an efficiently computable lower bound on the concurrence^30^ *C*. Let *i* and *j* (*k* and *l*) enumerate the computational basis vectors of the first (second) partition of the Hilbert space. Define $A := 4 \sum _{i<j, k<l} | ikjl \rangle -| jkil \rangle -| iljk \rangle +| jlik \rangle \times h.c.$ and let a state be represented in its spectral composition $\rho = \sum _i \mu _i | \Psi _i \rangle \langle \Psi _i |$. With a dominant eigenvector $| \Psi _0 \rangle$, define $| \xi \rangle \propto A | \Psi _0 \rangle \otimes | \Psi _0 \rangle$ and

$$\begin{aligned} T_{i,j}:= \sqrt{\mu _i \mu _j} \langle \Psi _i | \otimes \langle \Psi _j | | \xi \rangle . \end{aligned}$$

The quasipure concurrence is defined by the singular values $S_i$ of the matrix $T_{i,j}$

$$\begin{aligned} C_{qp}(\rho ):=S_0 - \sum _{i>0}S_i \le C(\rho ) \end{aligned}$$

and detects entanglement for positive values. We have $C_{qp}(\rho ) = C(\rho )$ for two qubits. In Fig. 3 we plotted directly $C(\rho )$ including the negative values.
